# Citrus Plants: A Model System for Unlocking the Secrets of NO and ROS-Inspired Priming Against Salinity and Drought

**DOI:** 10.3389/fpls.2016.00229

**Published:** 2016-02-26

**Authors:** Athanassios Molassiotis, Dominique Job, Vasileios Ziogas, Georgia Tanou

**Affiliations:** ^1^Faculty of Agriculture, Aristotle University of ThessalonikiThessaloniki, Greece; ^2^AgroParisTech, Chair of Plant PhysiologyParis, France; ^3^CNRS/UCBL/INSA/Bayer CropScience Joint Laboratory-UMR5240Lyon, France

**Keywords:** abiotic stress, drought, priming, proteins, salinity

## Abstract

Plants treated with chemical compounds can develop an enhanced capacity to resist long after being subjected to (a)biotic stress, a phenomenon known as priming. Evidence suggests that reactive oxygen species (ROS) and reactive nitrogen species (RNS) coordinately regulate plant stress responses to adverse environmental conditions; however, the mechanisms underlying this function remain unknown. Based on the observation that pre-exposure of citrus (*Citrus aurantium* L.) roots to the NO donor sodium nitroprusside (SNP) or to H_2_O_2_ prior to NaCl application can induce acclimation against subsequent stress we characterized the changes occurring in primed citrus tissues using several approaches. Herein, using this experimental model system, we provide an overview of our current knowledge of the possible mechanisms associated with NO and H_2_O_2_ priming to abiotic stresses, particularly concerning salinity and drought. The data and ideas presented here introduce six aspects of priming behavior in citrus under abiotic stress that provide knowledge necessary to exploit priming syndrome in the context of sustainable agriculture.

## Introduction

Environmental stress factors, such as drought and salinity, strongly affect plant growth and pose a growing threat to sustainable agriculture (Golldack et al., 2014). This has become a hot issue due to concerns about the effects of climate change on plant resources, biodiversity and global food security (Ahuja et al., 2010). Consequently, understanding the mechanisms underlying plant abiotic stress acclimation helps us to develop fruitful new agricultural strategies (Beckers and Conrath, 2007). Evidence suggests that plants are capable of inducing some stress “memory,” or “stress imprinting” following a first stress exposure that leads to acclimation to a later (a)biotic stress. Through priming (also known as hardening), plants are able to induce responses to a range of stresses, providing low-cost protection in relatively high stress-pressure conditions (Borges et al., 2014). Despite priming phenomena have previously been widely described under biotic stress (Prime-A-Plant Group et al., 2006; Conrath et al., 2015) and in the invigoration of seeds (Rajjou et al., 2012), the mechanisms of long-lasting priming are still unclear, notably under abiotic stress (Tanou et al., 2012b). It has been suggested that hormone-dependent pathways and availability of signal transduction proteins along with epigenetic mechanisms, such as histone modifications and DNA methylation, are involved in priming against abiotic stress (Bruce et al., 2007; Conrath, 2011). Recently, Jiménez-Arias et al. (2015) showed that *Arabidopsis* seed-based priming against salt stress involves epigenetic changes (DNA hypomethylation) in genes controlling proline metabolism. In this regard, it has been proposed that priming stimulates salicylic acid (SA), abscisic acid (ABA), and jasmonic acid (JA) signaling that could facilitate the transcriptional induction of defense genes and epigenetic changes; this *trans*-generational induced resistance is elicited by the RNA-directed DNA methylation (RdDM) pathway, which triggers heritable changes in DNA methylation that can direct priming-inducing chromatin modifications at defense gene promoters (Pastor et al., 2013).

Almost all abiotic stressors generate reactive oxygen species (ROS) and reactive nitrogen species (RNS), resulting in oxidative and nitrosative stress in plants (Molassiotis and Fotopoulos, 2011). It has been increasingly evident that RNS (in the form of nitric oxide, NO) and ROS (in the form of H_2_O_2_) play important roles in priming phenomena in various annual plants (Del Río, 2015). Nevertheless, a priming approach has a great potential if studied in non-model long-lived fruit trees species (Molassiotis et al., 2010). Citrus is widely cultivated in Mediterranean-type ecosystems where climatic change is expected to amplify drought and salinity stress (Gordo and Sanz, 2010). Basal resistance by itself is too weak to protect citrus against environmental stimuli, since it is sensitive to oxidative and nitrosative stress induced by various abiotic stress conditions (Ziogas et al., 2013). Hence, the possible implications of NO and H_2_O_2_ in the acclimation of citrus plants to adverse environmental conditions, as well as the interactions between the two molecules, were studied. In this regard, we initially documented at physiological level that NO and H_2_O_2_ are able to induce priming against salt stress by pre-treating the roots of sour orange (*Citrus aurantium* L.) seedlings either with the NO donor sodium nitroprusside (SNP) or with H_2_O_2_ prior to NaCl application (see Tanou et al., 2009b). Using this experimental system, the mechanisms by which citrus plants respond to salinity were investigated in order to gain a wide understanding of oxidative- and nitrosative-associated priming in plants. In this review, we summarize our current knowledge of the possible mechanisms associated with NO- and H_2_O_2_-induced salinity and drought acclimation in citrus plants. Overall, this approach reveals the following six aspects regarding the mechanism of NO- and H_2_O_2_-associated priming events in citrus against abiotic challenges.

## NO and H_2_O_2_-Originated Priming is Associated with Induced Antioxidant Activity

One of the mechanisms actively employed by primed plants to survive under abiotic stress is the induction of the antioxidant defense system (Hossain et al., 2015). In citrus plants it was evidenced that pre-treatments with NO and H_2_O_2_ prior to NaCl stress induced antioxidative defense-related enzymatic activity [e.g., superoxide dismutase (SOD), catalase (CAT), ascorbate peroxidase (APX), and glutathione reductase (GR)] (Tanou et al., 2009b) and mRNA expression [(eg., Cu/Zn-SOD, Mn-SOD and Fe-SOD, xanthine oxidoreductase (XO), and alternative oxidase (AOX)] (Tanou et al., 2012a). Another antioxidant function of NO arises from the observation that NO protects citrus leaves from DNA strand cleavage caused by hydroxyl radical ^•^OH produced following NaCl application (Tanou et al., 2009b), thereby avoiding oxidative damage induced by most harmful ROS. It has been proposed that NO protects from ^•^OH-stimulated oxidative stress in two possible ways: directly by scavenging ROS, and indirectly by NO-mediated induction of ferritin proteins that contributes to diminishing free-Fe^2+^ levels and Fenton-type reactions within the organelles (Martin et al., 2009). It had been demonstrated that ^•^OH effectively targeted DNA methylation and regulatory genes (Shen et al., 2014), implying that NO may have an important role in priming process, at least in part, by DNA methylation-based epigenetic modifications.

## The Priming Action of NO and ROS is Long–Distance and Long-Lasting

Systemic signals are perceived in distant plant tissues and initiate systemic stress responses through priming (Gaupels and Corina Vlot, 2012). Knowledge on such long-distance signaling has been recently documented in various plant systems (Frost et al., 2008; Chaturvedi et al., 2012; Shah et al., 2014). According to the above studies, phloem is the likely path for systemic transmission or movement of signals associated with the acclimation process (Chaturvedi et al., 2012; Ruiz-Medrano et al., 2012). Similarly, root-applied NO or H_2_O_2_ remarkably increased NO and H_2_O_2_ steady-level in the leaves of citrus, indicating that these two molecules are systemic priming elicitors at the whole-plant level (Tanou et al., 2012a). Histochemical localization of H_2_O_2_ and 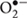 production in citrus leaves using 3,3′-diaminobenzidine (DAB) and nitroblue tetrazolium (NBT) staining indicated that ROS specifically accumulated in the vicinity of the primary vein (Tanou et al., 2012a). Such preferentially topological distribution of ROS reflects the greater exposure of periveinal cells to systemic signals, and that, when diffusing out of the veins, the concentration is diluted and therefore the cells near the vascular bundles are more likely to react (Alvarez et al., 1998). Further evidence for the long-distance and long-lasting nature of NO and H_2_O_2_ arose from the observation that both NO and H_2_O_2_-derived 4,5-diaminofluorescin diacetate (DAF-2DA) and 2,′7′-dichlorofluorescin diacetate (DCF-DA) fluorescence were detected in the vascular tissues (xylem and particularly phloem) and in the upper and lower epidermal cells under normal and NaCl stress conditions and occurred following 8 days of NO/H_2_O_2_ application (Tanou et al., 2012a). In citrus we also observed that the local application of NO in roots induced drought acclimation in leaves for at least 35 days after exposure to PEG stress (Ziogas et al., 2015) further suggesting that NO priming signal is memorized and systemically transduced. However, the evidence for *in planta* NO and H_2_O_2_ long-lasting signaling is not strong enough to conclude their priming roles due to the short half-lives of NO and H_2_O_2_ (Neill et al., 2002). One mode of long-distance/lasting NO and H_2_O_2_ action may be associated with the auto-propagation of ROS and RNS waves throughout the plant (Mittler et al., 2011; Molassiotis and Fotopoulos, 2011). The initial abiotic stress-induced burst of ROS/RNS in a local group of plant cells triggers cell to cell communication that propagates throughout different tissues of the plant and carries a systemic signal over long distances (Miller et al., 2009). Another scenario might be the systemic NO and H_2_O_2_ signaling through binding to specific stress-related enzymes, such as mitogen-activated protein kinases (MAPKs) (Mishra et al., 2006) and *S*-nitrosoglutathione reductase (GSNOR) (Leterrier et al., 2011), thereby modifying their activity. In this regard, GSNOR transcripts in leaves and roots of salt-primed citrus plants were down-regulated showing that GSNOR could be considered a mechanism by which NO and H_2_O_2_ orchestrate priming signaling (Tanou et al., 2012a).

## Protein Reprogramming is a Key Mechanism of NO and H_2_O_2_-Induced Priming

Changes in environmental conditions are likely to cause rapid changes in the level, composition, and structure of different metabolites, proteins, and RNA molecules that precede signal transduction or stress acclimation events in plants (Baxter et al., 2014). An interesting finding that emerged from the work in citrus is the fact that the NaCl-responsive leaf proteome (85 proteins) was remarkably affected by pre-exposure to NO or to H_2_O_2_. Indeed, NO or H_2_O_2_ pretreatment prior to salt stress imposition, reversed a large part of the NaCl-responsive proteins (53 and 55 proteins, respectively; Tanou et al., 2009a). The major set of these proteins (46.7%) participate in photosynthesis and particularly in the Calvin cycle (e.g., several isoforms of Rubisco activase, Rubisco large subunit, fructose 1,6-bisphosphate aldolase, phosphoglycerate kinase, glyceraldehyde-3-phosphate dehydrogenase, phosphoribulokinase, transketolase, and carbonic anhydrase; Tanou et al., 2012a), this being a probable attempt at sustaining the photosynthesis rate during salt stress. Regulatory mechanisms of photosynthetic proteins were also studied in other stress-affected plant species and crops. For example, Komatsu et al. (2014) pointed the major proteomic alternation in wheat under various abiotic stresses focused on photosynthesis-responsive proteins. In addition to NO- or to H_2_O_2_-mediated regulation of protein expression during salinity acclimation, a more recent study showed that pre-treatment with NO also modulates specifically several PEG-affected proteins (e.g., glycolate oxidase, NADP-isocitrate dehydrogenase, and UPF0603 protein Atlg54780) in citrus plants experiencing priming against drought (Ziogas et al., 2015). These findings indicate that the priming function of NO and H_2_O_2_ in citrus plants is a dynamic, photosynthetic activity-demanding process that might be, at least partially, attributed to proteome reprogramming.

## NO and H_2_O_2_ -Altered Protein Posttranslational Modification Physiognomy

It has been well documented that ROS and RNS exposure can prime plants against abiotic stresses through chemical reactions with specific target proteins that result in covalent posttranslational protein modifications (PTMs), altering protein function and activity (Lounifi et al., 2013). Therefore, the characterization of PTMs is crucial for a deeper understanding of NO and H_2_O_2_ priming. Particularly, protein carbonylation is a type of protein oxidation driven by oxidative stress, which occurs by direct attack on Lys, Arg, Pro, or Thr protein residues (Oracz et al., 2007). It has been shown that basal levels of protein carbonylation under control conditions increase sharply when citrus plants are grown under salinity (Tanou et al., 2009a, 2012a), inhibiting enzyme activities and increasing protein susceptibility toward proteolytic attack (Dunlop et al., 2002). Proteomic 2DE-OxyBlot-based analysis in citrus indicated that H_2_O_2_ and SNP pretreatments before salt stress prevented the NaCl-induced protein carbonylation to the levels of untreated control plants and allowed identifying 40 carbonylated proteins showing a reversal in accumulation level upon H_2_O_2_ or SNP application (Tanou et al., 2009a). Furthermore, tyrosine (Tyr) nitration, i.e., the addition of a nitro group (NO_2_) to one of the two equivalent ortho carbons of the aromatic ring of Tyr residues in the presence of excess levels of ROS and NO or NO-derived species, is recognized as an important redox PTM (Corpas et al., 2009). Protein Tyr nitration has been established as a biomarker of systemic “nitroxidative stress,” leading plant metabolism to a pro-oxidant status that disrupts NO signaling and induces protein structural and functional changes, some of which contribute to altered cell and tissue homeostasis (Corpas et al., 2013). Similar to the pattern of protein carbonylation, Tyr-nitration increased in citrus leaves exposed to salinity or to NO or H_2_O_2_ under stress-free conditions but diminished to control basal levels when these chemical treatments were applied before the imposition of NaCl stress (Tanou et al., 2012a). These results clearly show that citrus plants adopt a common oxy- and nitro-based stress-alleviating mechanism in their leaves. In both cases the results strengthen the notion that these PTMs are not just a ‘fingerprint’ of oxidative and nitrosative stress, but also they are essential components of citrus priming mechanism.

*S*-nitrosylation is another PTM that has been validated as signaling mechanism mediated by nitrosative/oxidative stress that occurs on cysteine (Cys) residues, being redox reversible with high spatial and temporal specificity modifying protein activity and accumulation (Astier and Lindermayr, 2012). In citrus leaves subjected to NaCl, *S*-nitrosylation decreased whereas NO or H_2_O_2_ pre-treatments before salt stress substantially increased protein *S*-nitrosylation. Remarkably, this response was in contrast to protein carbonylation and Tyr-nitration patterns under the same experimental conditions (Tanou et al., 2009a, 2012a), denoting differences of PTMs regulation during salinity acclimation. By studying the ROS and RNS priming input against salinity we performed a comparative analysis of carbonylated, nitrated and nitrosylated proteome in citrus plants (Tanou et al., 2012a). This approach revealed that the majority of the PTM-targeted proteins in leaves were involved in Calvin–Benson cycle followed by disease/defense mechanisms and protein destination. More interestingly, among the 92 carbonylated, 88 Try-nitrated and 82 *S*-nitrosylated proteins, approximately one third of them, namely 34, 26 and 36 proteins respectively were specifically carbonylated or Try-nitrated or *S*-nitrosylated (Tanou et al., 2012a). On the contrary, 22 citrus proteins, including Rubisco large subunit, GAPDH subunits A, B, the photosystem II 44 kDa reaction center, sedoheptulose-1,7-bisphosphatase, phosphoribulokinase, carbonic anhydrase, light-harvesting chlorophyll a/b binding protein, ribulose-5-phosphate 3-epimerase, fructose-1,6-bisphosphate aldolase, glycolate oxidase and sinapyl alcohol dehydrogenase, simultaneously targeted by these three PTMs (Tanou et al., 2012a). It is also interesting that in a previous study we observed an overlap (17 proteins) among the NO– or H_2_O_2_–targeted carbonylated (*n* = 40) and *S*-nitrosylated (*n* = 49) proteins in citrus leaves experiencing priming effects against salinity (Tanou et al., 2009a), thereby disclosing that these proteins are common markers of NO and H_2_O_2_ signaling. Possible explanations for the co-modulated pattern of protein carbonylation and Tyr-nitration in contrast to *S*-nitrosylation have been proposed. The co-occurrence of more than one PTMs could be explained by the fact that the first PTM allosterically triggers the occurrence of other(s), thereby fine-tuning the degradation of damaged proteins by the proteasomes and/or offering protection against irreversible damage (Lounifi et al., 2013). It is therefore possible that *S*-nitrosylation might prevent the irreversible loss of function of proteins by protein oxidation due to carbonylation and/or Tyr-nitration by locking the structure of these proteins in a state under which they are no more sensitive to ROS/RNS attack (Sun et al., 2006). Altogether, the results support the existence of a link between oxidative and nitrosative regulatory events through PTMs characterizing priming phenomena in citrus plants.

## Leaves and Roots Exhibit Both Common and Distinct Priming Codes in Response to NO and H_2_O_2_

When examining the expression of genes involved in NO production [e.g., nitric oxide synthase (NOS)-like proteins, nitrate reductase (NR), nitrite reductase (NiR)] as well as H_2_O_2_ generation [e.g., diamine oxidases (DAO) and polyamine oxidases (PAO)] we observed a distinctive complementary pattern in leaves and roots of citrus exposed to NaCl (Tanou et al., 2012a), likely reflecting locally and systemically differences in oxy/nitro priming signaling. Consistent with these tissue-specific transcriptional patterns, we also uncovered the existence of a tissue-dependent regulation of *S*-nitrosylation induced by NO and H_2_O_2_ stimuli (Tanou et al., 2012a). The *S*-nitrosylation was induced in leaves and depressed in roots following NO and H_2_O_2_ priming treatments, which is in agreement with the differential spatial distribution of oxidative and nitrosative stress in leaves versus roots documented in *Lotus japonicus* (Signorelli et al., 2013). By contrast, chemical pre-treatments with NO or H_2_O_2_ suppressed protein carbonylation and nitration in both leaves and roots exposed to NaCl, suggesting that some PTM responses are commonly regulated in the different types of citrus tissues (Tanou et al., 2012a). This is further strengthened by the fact that SNP stimulated Tyr-nitration in leaves and roots during acclimation to drought stress (Ziogas et al., 2015). Such functional differences between leaves and roots may be partially attributed to the tissue differences in the types of NO and ROS generation systems. Superoxide (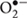) and H_2_O_2_ produced through SODs up-regulation and polyamine degradation in roots of citrus just following pre-treatments with NO or H_2_O_2_ can activate specific signaling pathways distinct from those perceived by leaves (Tanou et al., 2012a). Analogously, NO accumulation via NR activation in citrus leaves subjected to NaCl or PEG stress (Tanou et al., 2012a) accompanies several different NO-signaling events which could regulate downstream pathways and stress acclimation (Baudouin and Hancock, 2014).

## Signaling Cross-Talk Between NO/ROS and Other Pathways

Recent studies have reported that many abiotic stress responses are coordinated by various signaling networks, particularly involving phytohormones, ROS, and RNS (Considine et al., 2015). In NaCl-treated citrus, NO and ROS accumulation in local and systemic tissues showed considerable overlap whereas a large part of the NaCl-sensitive proteins were commonly modulated by NO and H_2_O_2_ (Tanou et al., 2009a), suggesting that there is a dynamic interplay between the signals regulating priming. It is noted that comparative proteomic analysis in citrus leaves under physiological non-stressful conditions revealed (i) an interlinked NO- and H_2_O_2_-modulated protein network, (ii) the carbonylation status of a very large portion of the carbonylated citrus mitochondrial proteins, which remained constant or depressed by NO and H_2_O_2_ (Tanou et al., 2010), disclosing the parallels in action between NO and ROS. Here, we also provide examples demonstrating that NO could interact with other signaling pathways in modulating stress acclimation. For example, we observed temporal–spatial interactions between NO-specific PTMs and polyamines (PAs) homeostasis/metabolism in citrus challenged with salinity (Tanou et al., 2014), thus confirming that NO and PAs displayed some overlapping functions in plants (Parra-Lobato and Gomez-Jimenez, 2011). Targeted analysis of PA-affected *S*-nitrosylated citrus proteins led us to propose that PAs binding to specific proteins, such as dehydroascorbate reductase (DHAR) and monodehydroascorbate reductase (MDHAR), may affect protein conformational parameters and also the environment surrounding Cys residues of protein targets (Tanou et al., 2014). Such chemical modifications of Cys residues could lead either to Cys oxidation through ROS or to *S*-nitrosylation through NO. Drought stress can induce accumulation of hydrogen sulfide (H_2_S) and its biosynthetic enzyme L-cysteine desulfhydrase (LCD) in citrus. Moreover, in citrus roots H_2_S can induce the expression of NiR and mitochondrial NAD(P)H dehydrogenases that are involved in NO production (Gupta et al., 2011) while H_2_S accumulation acts downstream of NO in PEG-induced *S-*nitrosylation (Ziogas et al., 2015). Meanwhile, drought stress induction of ABA accumulation is a well-known fact (Tan et al., 2003). In citrus the fact that PEG stress-induced ABA accumulation and the expression of 9-*cis*-epoxycarotenoid dioxygenase (NCED), a key enzyme in ABA biosynthesis, were depressed in plants pre-exposed to NO or to H_2_S (Ziogas et al., 2015) suggests that the interplay among NO, H_2_S, and ABA could be considered a mechanism by which citrus orchestrates drought stress acclimation.

## Conclusion and Future Perspectives

**FIGURE 1:**
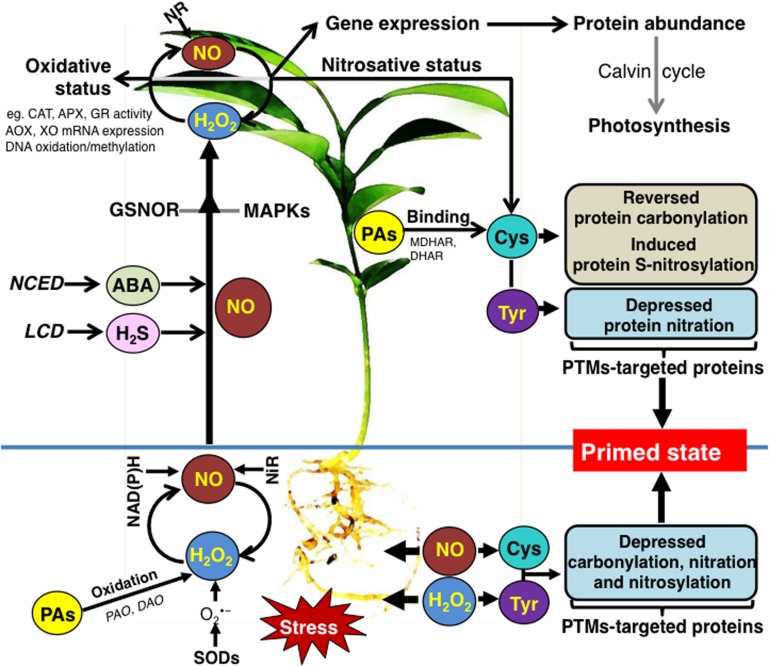
**Schematic overview of the signaling networks involved in the NO- and ROS-induced priming against salininy and drought stress in citrus plants (see text for details).** ABA, abscisic acid; AOX, alternative oxidase; APX, ascorbate peroxidise; CAT, catalase; Cys, cysteine; DAO, diamine oxidase; DHAR, dehydroascorbate reductase; GR, glutathione reductase; GSNOR, *S*-nitrosoglutathione reductase; H_2_S, hydrogen sulfide; LCD, L-cysteine desulfhydrase; MAPKs, mitogen-activated protein kinases; MDHAR, monodehydroascorbate reductase; NAD(P)H, mitochondrial NAD(P)H dehydrogenases; NCED, 9-*cis*-epoxycarotenoid dioxygenase; NiR, nitrite reductase; NR, nitrate reductase; PAO, polyamine oxidase; PTMs, posttranslational modifications; PAs, polyamines; SOD, superoxide dismutase; Tyr, tyrosine; XO, xanthine oxidoreductase.

Research performed over the last years documented that NO and H_2_O_2_ induce priming toward salinity and drought in citrus plants and most importantly reveals key aspects of this phenomenon. Based on these results, we propose a signaling network through which NO and H_2_O_2_ provoke priming responses in leaves and roots of citrus (**Figure 1**). While several components of the priming mechanism have been proposed, we still lack a thorough understanding of the complex mode of action of specific signaling molecules in plant stress acclimation. In this regard, various -omics techniques investigating both roots and leaves should be combined to fully understand NO and ROS-induced priming. It will be perhaps the major challenge of priming research to test these chemical agents against multiple abiotic stresses that occur in field conditions. Such an approach would allow establishing priming technology as a tool to manage crop yield.

## Author Contributions

AM, DJ, VZ, and GT wrote the paper.

## Conflict of Interest Statement

The authors declare that the research was conducted in the absence of any commercial or financial relationships that could be construed as a potential conflict of interest.
